# End of life breast cancer care in women with severe mental illnesses

**DOI:** 10.1038/s41598-021-89726-y

**Published:** 2021-05-13

**Authors:** Guillaume Fond, Vanessa Pauly, Audrey Duba, Sebastien Salas, Marie Viprey, Karine Baumstarck, Veronica Orleans, Pierre-Michel Llorca, Christophe Lancon, Pascal Auquier, Laurent Boyer

**Affiliations:** 1 Faculté de Médecine - Secteur Timone, EA 3279: CEReSS -Centre d’Etude et de Recherche sur les Services de Santé et la Qualité de vie, Aix-Marseille Univ., 27 Boulevard Jean Moulin, 13005 Marseille, France; 2 Department of Epidemiology and Health Economics, APHM, Marseille, France; 3 Department of Medical Information, APHM, Marseille, France; 4 Department of Adult Oncology, APHM, Marseille, France; 5 CHU Clermont-Ferrand, Clermont-Ferrand, France; 6 Department of Psychiatry, APHM, Marseille, France

**Keywords:** Public health, Breast cancer

## Abstract

Little is known on the end-of-life (EOL) care of terminal breast cancer in women with severe psychiatric disorder (SPD). The objective was to determine if women with SPD and terminal breast cancer received the same palliative and high-intensity care during their end-of-life than women without SPD. *Study design, setting, participants*. This population-based cohort study included all women aged 15 and older who died from breast cancer in hospitals in France (2014–2018). *Key measurements/outcomes*. Indicators of palliative care and high-intensity EOL care. Multivariable models were performed, adjusted for age at death, year of death, social deprivation, duration between cancer diagnosis and death, metastases, comorbidity, smoking addiction and hospital category. The analysis included 1742 women with SPD (287 with bipolar disorder, 1075 with major depression and 380 with schizophrenia) and 36,870 women without SPD. In multivariate analyses, women with SPD had more palliative care (adjusted odd ratio aOR 1.320, 95%CI [1.153–1.511], *p* < 0.001), longer palliative care follow-up before death (adjusted beta = 1.456, 95%CI (1.357–1.555), *p* < 0.001), less chemotherapy, surgery, imaging/endoscopy, and admission in emergency department and intensive care unit. Among women with SPD, women with bipolar disorders and schizophrenia died 5 years younger than those with recurrent major depression. The survival time was also shortened in women with schizophrenia. Despite more palliative care and less high-intensity care in women with SPD, our findings also suggest the existence of health disparities in women with bipolar disorders and schizophrenia compared to women with recurrent major depression and without SPD. Targeted interventions may be needed for women with bipolar disorders and schizophrenia to prevent these health disparities.

## Introduction

Severe psychiatric disorders (SPD) including schizophrenia, bipolar disorder and recurrent major depression are an increasing burden in the Western countries. Patients with SPD are at increased risk for comorbid chronic physical conditions including cancer^1^. With 13.4% of all cancers, breast cancer was the leading cancer among women in 40 European countries and in 16.2% the leading cause of cancer death among European women according to a study published in 2018^2^. Higher breast cancer incidence and mortality have been documented women with SPD compared with their non-SPD counterparts^3^. This increased mortality may be explained by different care of cancer at every stage including screening, care and end-of-life (EOL)/terminal cancer. Previously, we have found that men and women with SPD died younger from their terminal cancer compared to non-SPD (by 8 years for schizophrenia^4^, 5 years for bipolar disorders^5^ and 3 years for recurrent major depressive disorder^6^). Women with SPD have a loss of chance of being screened for breast cancer^7^. For example, women with schizophrenia may deny the cancer symptoms and be diagnosed with high-stage disease at diagnosis^8^. After diagnosis, women with SPD may not receive the same cancer care than women without SPD because of their lower awareness and understanding of the disease and decreased cooperation with medical staff^9,10^. A retrospective American cohort study including 16,636 women found that women with SPD had an increased risk of more than 36% delay in initial treatment of ≥ 60 days from diagnosis^11^. In addition, women with SPD are a population largely neglected in health disparities work, which have some important specificities compared to men that justify a targeted work. For example, women with schizophrenia have a later age at illness onset and are more frequently married with children compared to men with schizophrenia^12^. On the contrary, they have a more severe cognitive impairment that may impact the EOL cancer care decisions^12^. Women are at a 2 to 3 increased risk of recurrent major depression compared to men. Depression may strongly impact the cancer care through impaired motivation, increased psychic suffering and desire to die^13^. Women have several good prognosis factors: they are more compliant into treatment with less hostile behavior and are less frequently smokers compared to men^14^. For all these reasons, a work targeting women with SPD is justified and can provide new information compared to an approach including both men and women.

To date, no data are available on the EOL care of women with SPD and terminal breast cancer. Because of gender disparities in health care use and access to the disadvantage of women^15^, it appears necessary to explore EOL care in women with cancer to complete previous works including men and women. Palliative care and the withdrawal of high-intensity care are recommended in the EOL period of women with terminal breast cancer by the National Comprehensive Cancer Network (NCCN) and the American Society of Clinical Oncology (ASCO)^16^. Palliative care improves the quality of life of patients and their families who are facing terminal cancer issues. Its aim is to prevent and relieve suffering through the early identification, correct assessment and treatment of physical and psychological pain and other physical, psychosocial or spiritual issues^17^.

The objective was to determine if women with SPD and terminal breast cancer received the same palliative and high-intensity care during their end-of-life than women without SPD.

## Methods

### Study design and data source

A population-based cohort study was carried out using the French national hospital database (Programme de Médicalisation des Systèmes d’Information). This study was carried out following the RECORDs and STROBE reporting guidelines (http://www.equator-network.org/). The database contains anonymized information prospectively collected from all public and private hospitals in France for acute and psychiatric hospitalizations. Inpatient stays are converted into single diagnosis-related groups based on standard discharge abstracts containing administrative information and clinical information: primary/secondary diagnoses, using the International Classification of Diseases, Tenth Revision (ICD-10), as well as procedural codes associated with the care provided. The PMSI database is used to determine financial resources, and is frequently and thoroughly verified by both its producer and the paying party, with possible financial and legal consequences. This database is of an acceptable quality, taking into account the natural limits to precision imposed by the methodology and terminologies used to code conditions and procedures^18^.

Since the study was strictly observational and used anonymous data, in accordance to the laws that regulate “non-interventional clinical research” in France, the written informed consent from the participants or the authorization from any other ethics committee were not required to conduct this study.

### Study population

We included all women aged 15 and older who died from breast cancer in the hospital in France between January 1, 2014, and December 31, 2018. Women with breast cancer were identified using a French validated algorithm developed by the French National Institute of Cancer specifically designed to identify cancer-related treatment in the French national hospital database^19^. This algorithm relies on multiple steps of selection involving ICD-10 codes related to breast cancer (C50*, D05*, D486) and medical and surgery acts related to breast cancer support (e.g., breast ablation, breast reconstruction for example). Then, women with breast cancer were included if they had at least one end-of-life inclusion criteria identified in the last three months of life: diagnosis of metastatic stage (ICD-10 codes C78 and C79) or hospitalization into a palliative unit or bed care or ICD-10 code for palliative care (Z515)^20,21^.

The group “women with SPD” was defined by women with at least one diagnostic of bipolar disorder (ICD codes F30*, F31*) OR “recurrent major depression” (F33*) OR “schizophrenia” (F20*, F22*, F25*) in the PMSI-MCO database and/or in the PMSI–PSY database during the 4-years period before death.

The group “women without SPD” included all women excluding women with diagnosis of SPD in the acute and/or psychiatric hospitalizations databases during the 4-years period before death.

We extracted and computed the following demographic and clinical women’ characteristics from the database:

- Age at death;
- Social deprivation assessed by an index validated on French data and based on the postal code of the domicile^22^. The social deprivation index involves four socioeconomic ecological variables: percentage of high-school graduates, median household income, percentage of blue-collar workers and the unemployment rate. The social deprivation index was categorized according to quartiles, from the least (Q1) to the most deprived (Q4);
- Year of death;
- Duration from cancer diagnosis (first date of hospitalization with cancer diagnosis since 2011) to death;
- Metastasis (yes or no) (ICD-10 codes C78 and C79 recorded as primary or secondary diagnoses in the last 3 months of life), as a metastasis diagnosis may accelerate palliative care admission;
- Non-cancer comorbidities assessed using the Charlson modified Comorbidity Index^23^ (computed from ICD-10 codes recorded as primary or secondary diagnoses over the course of the last 12 months of life, excluding the 2 items referring to cancer, i.e., metastatic solid tumor and malignancy);
- Smoking addiction (yes or no) (ICD-10 codes F17* recorded as primary or secondary diagnoses in the last 12 months of life);
- Hospital category(specialized cancer center or non-specialized center) (at last hospitalization before death).

The ICD-10 codes were validated by two independent expert coders (from the department of medical information). Any discrepancies were resolved by consensus with a third expert coders from the department of medical information.

### Outcome measures

The outcome measures corresponded to the palliative care and the high-intensity end-of-life care indicators, based on previously defined criteria^24,25^. The palliative care indicators included access to palliative care in the last 31 days and duration in days between the first palliative care and death. The high-intensity end-of-life care indicators included: intrahospital chemotherapy in the last 14 days of life, mechanical ventilation, blood transfusion, surgery, imaging or endoscopy, at least one emergency department (ED) or intensive care unit (ICU) admission, and more than one admission in acute care unit in the last 31 days of life. All variables were binary (*i.e.,*, yes or no), except for the duration in days between the first palliative care and death, which was a continuous variable. Appendix A1 lists the specific codes used for each outcome.

### Ethical concerns

Because this study was strictly observational and based on anonymous data, the written informed consent from the participants or the authorization from an ethical committee for dealing with human issues was not required in accordance with the French laws.

### Statistical analysis

Comparisons between women with SPD and women without SPD were performed for sociodemographic, clinical and hospital data using univariate generalized linear models (with logit function for binary outcomes and log-normal distribution for continuous outcomes) using the hospital as a random intercept to take into account correlation of patient’s characteristics among hospitals.

We also performed comparisons of subgroups (bipolar disorder vs. recurrent major depression vs. schizophrenia).

Then, we performed as many multivariate analyses as outcomes to analyze the association between the groups (i.e., women with and without SPD) and each outcome. Binary outcomes were analyzed using a multivariable generalized linear model (logit function) with the hospital as a random intercept to take into account correlation of patient’s characteristics among hospitals and to measure subject-specific effects. For continuous data (non-Gaussian distribution), a multivariable generalized log-linear model with the hospital as a random intercept was used to take into account correlation of patient’s characteristics among hospitals and to measure subject-specific effects. The following confounding factors were identified based on previous works on end-of-life care^4^ and included in the models: age at death (continuous variable), year of death (five categories: 2014, 2015, 2016, 2017, 2018), social deprivation (four categories/quartiles from the least (Q1) to the most deprived (Q4)), duration between cancer diagnosis and death (days), metastases (yes/no), Charlson modified comorbidity index (three categories: 0, 1 or 2, >  = 3 comorbidities), smoking addiction (yes/no) and hospital category (two categories: specialized for cancer vs. non-specialized centers).

The statistical analysis was performed with SAS 9.4 (SAS Institute) using proc glimmix. Statistical significance was defined as *p* < 0.05. Multiple comparison corrections based on the false discovery rate method were applied.

## Results

### Characteristics of the patients

**Figure 1:**
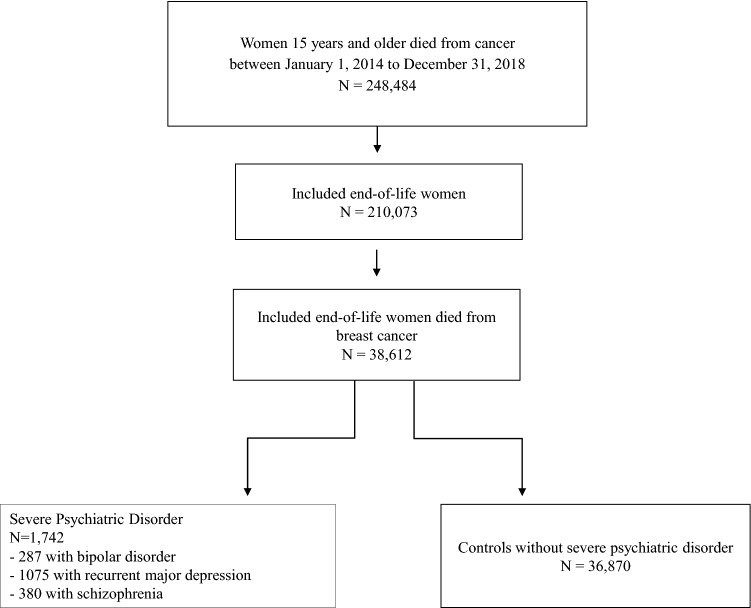
Flow chart.

Overall, 248,484 women aged 15 and more who died from cancer between 2014 and 2018 were identified in France. Among them, 210,073 were identified with terminal/ EOL cancer and 38,612 had breast cancer. A total of 1742 women with SPD (380 with schizophrenia, 287 with bipolar disorder and 1075 with recurrent major depression) and 36,870 controls without SPD were included in the analyses (Fig. 1).

Patient characteristics are described in Table 1 and the differences between bipolar disorder, recurrent major depression and schizophrenia are presented in the supplementary Table 1. The median age at death was similar between women SPD and without SPD but markedly lower in respectively schizophrenia and bipolar disorder compared to recurrent major depression.

**Table 1:** Characteristics of the 1742 women who died from their terminal breast cancer between 2014 and 2018 in France with diagnosis of severe psychiatric disorder* (SPD) compared to the 36,870 controls without SPD.

	Controls (N = 36,870)		SPD women (N = 1742)		*p* value
	N	%	N	%	
Age at death, years (mean [SD])	**67.8**	**[14.7]**	**67.4**	**[14.1]**	0.3170
Social deprivation index					0.0592
More favored (Q1)	9664	26.2	504	28.9	
Favored (Q2)	6877	18.7	329	18.9	
Deprived (Q3)	11,247	30.5	507	29.1	
More deprived (Q4)	9082	24.6	402	23.1	
Year of death					0.1077
2014	7140	19.4	308	17.7	
2015	7228	19.6	382	21.9	
2016	7339	19.9	347	19.9	
2017	7587	20.6	344	19.8	
2018	7576	20.6	361	20.7	
Survival time, days (median [IQR])	886.0	[327–1442]	918.0	[401–1438]	0.2102
Metastasis	**31,896**	**86.5**	**1422**	**81.6**	**< 0.001**
Smoking addiction	**972**	**2.6**	**130**	**7.5**	**< 0.001**
**Comorbidities**					
Charlson's comorbidity modified score					**< 0.001**
0	20,660	56.0	731	42.0	
1–2	10,805	29.3	622	35.7	
≥ 3	5405	14.7	389	22.3	
Renal disease	**2299**	**6.2**	**192**	**11.0**	**< 0.001**
Rheumatologic disease	**249**	**0.7**	**25**	**1.4**	**0.0002**
Peripheral Vascular disease	**994**	**2.7**	**87**	**5.0**	**< 0.001**
Peptic Ulcer disease	399	1.1	19	1.1	0.9732
Hemiplegia or Paraplegia	**2815**	**7.6**	**196**	**11.3**	**< 0.001**
Moderate or severe liver disease	1724	4.7	75	4.3	0.4734
Mild liver disease	**936**	**2.5**	**78**	**4.5**	**< 0.001**
AIDS/HIV	**56**	**0.2**	**7**	**0.4**	**0.023**
Diabetes with complications	**812**	**2.2**	**76**	**4.4**	**< 0.001**
Diabetes without complications	**4430**	**12.0**	**309**	**17.7**	**< 0.001**
Dementia	**1550**	**4.2**	**165**	**9.5**	**< 0.001**
Cerebrovascular disease	**1990**	**5.4**	**131**	**7.5**	**0.0001**
Chronic pulmonary disease	**1883**	**5.1**	**188**	**10.8**	**< 0.001**
Congestive Heart Failure	**4811**	**13.1**	**301**	**17.3**	**< 0.001**
Myocardial infarction	**1010**	**2.7**	**74**	**4.3**	**0.0002**
Hospital category (at last hospitalization before death)					**< 0.001**
Specialty Center	**9074**	**24.6**	**504**	**28.9**	
Nonspecialty Center	27,796	75.4	1238	71.1	

Significant results are in bold.

*N* number of patients, % percentage, *IQR* interquartile range, *Q* quartile (from Q1 to Q4), *SD* standard deviation.

*Defined by a diagnosis of recurrent major depression, bipolar disorder or schizophrenia on the study period.

Women with SPD were less frequently diagnosed with metastasis than women without SPD (with no difference between bipolar disorder, recurrent major depression and schizophrenia). The survival time was similar between SPD women and those without SPD but markedly lower in schizophrenia patients compared to recurrent major depression. Women with SPD had more frequently a smoking addiction diagnosis compared to women without SPD without significant differences between psychiatric diagnoses. Women with SPD had a higher Charlson index score, with more frequently renal disease, rheumatologic disease, peripheral vascular disease, hemiplegia or paraplegia, mild liver disease, AIDS/HIV, diabetes with or without complications, dementia, cerebrovascular disease, chronic pulmonary disease, congestive heart failure and myocardial infarction.

Women with SPD were more frequently hospitalized in specialized cancer center in their last month of life compared to women without SPD with no difference between bipolar disorder, recurrent major depression and schizophrenia.

### Multivariate analyses

Compared to women without SPD, women with SPD were found (Table 2) to have more frequent palliative care in the last 31 days of life (adjusted odd ratio aOR 1.320, 95%CI [1.153–1.511], *p* < 0.001) and longer palliative care follow-up before death (adjusted beta = 1.456, 95%CI (1.357–1.555), *p* < 0.001); to receive less chemotherapy in the last 14 days of life (aOR 0.703, CI95% [0.600–0.825], *p* < 0.001), less surgery (aOR 0.829, IC95% [0.703–0.976], *p* = 0.035) and less imaging/endoscopy (aOR 0.880, CI95% [0.787–0.984], *p* = 0.035) in the last 31 days of life; and to be less likely admitted in ED (aOR 0.846, CI95% [0.757–0.946], *p* = 0.009) and ICU (aOR 0.783, CI95% [0.637–0.962], *p* = 0.035) during the 31 days preceding death. No significant association was found for mechanical ventilation and blood transfusion in the last 31 days of life.

**Table 2:** Comparison of end of life palliative and high-intensity care of terminal breast cancer between women with and without SPD.

	Univariate analysis					Multivariate analysis*		
	Women without SPD (N = 36,870)		Women with SPD (N = 1742)		*p* value ***	aOR or Beta**	[CI95%]	adjusted *p* value***
	N or Median	% or [IQR 95%]	N or Median	% or [IQR 95%]				
**Palliative care**								
Palliative care in the last 31 days of life	27,706	75.2	1417	81.3	**< 0.001**	1.320	1.153–1.511	**< 0.001**
Duration (days) between the first palliative care and death (for patients with palliative care)	19	[8–44]	28	[11–83]	**< 0.001**	1.456	1.357–1.555	**< 0.001**
**High-intensity end-of-life care**								
Intrahospital chemotherapy in the last 14 days of life	5620	15.2	194	11.1	**< 0.001**	0.703	0.600–0.825	**< 0.001**
Mechanical ventilation in the last 31 days of life	3660	9.9	196	11.3	0.716	0.983	0.834–1.158	0.836
Blood transfusion in the last 31 days of life	4618	12.5	204	11.7	0.742	0.910	0.778–1.065	0.301
Surgery in the last 31 days of life	4410	12.0	179	10.3	0.144	0.829	0.703–0.976	**0.035**
Imaging/ Endoscopy in the last 31 days of life	26,045	70.6	1,151	66.1	0.194	0.880	0.787–0.984	**0.035**
At least one ED admission in the last 31 days of life	16,023	43.5	710	40.8	**0.016**	0.846	0.757–0.946	**0.009**
At least one ICU admission in the last 31 days of life	2564	7.0	117	6.7	0.384	0.783	0.637–0.962	**0.035**

*N* number of patients, % percentage, *aOR* adjusted odds ratio, CI95% 95% confidence level, *ED* emergency department, *ICU* intensive care unit, *IQR* interquartile range. Significant associations are in bold.

*Adjustment on the following confounding factors: age at death, social deprivation, year of death, survival time, metastases, Charlson modified comorbidity index, smoking addiction and hospital category.

**Beta and CI95% are issued from a log normal mixed analysis and are back transformed: for example, beta = 1.46 means that the duration between the first palliative care and death is 46% higher for patients with schizophrenia.

***THE false discovery rate (FDR) was applied.

*p*-value in bold: statistically significant.

The comparisons between bipolar disorder, recurrent major depression and schizophrenia are presented in the supplementary Table 2. Women with bipolar disorders and schizophrenia had more mechanical ventilation than women with recurrent major depression, and women with bipolar disorders had more surgery than women with recurrent major depression. No other difference was found between the three groups.

## Discussion

This nationwide database study including 38,612 women dying from their terminal breast cancer between 2014 and 2018 in France has revealed discrepancies between those with a diagnostic of SPD and those without. Women with SPD were found to receive less frequently a metastasis diagnosis, to have more smoking addiction and somatic comorbidities, and to be more frequently hospitalized in specialized cancer center. In multivariate analyses, women with SPD received more palliative care and less high-intensity care during their last month of life compared to women without SPD, especially less chemotherapy, surgery, imaging and less admission in ED/ICU units.

Overall, women with SPD may receive more appropriate EOL care as palliative care and the withdrawal of high-intensity care are recommended in the EOL period by the National Comprehensive Cancer Network (NCCN) and the American Society of Clinical Oncology (ASCO)^16^. However, our data shows that analyzing more precisely each psychiatric disorder revealed important disparities between women with recurrent major depression and those with bipolar disorders or schizophrenia. Women with bipolar disorders and schizophrenia died 5 years younger than those with recurrent major depression or those without SPD. In addition, the survival time was lower in schizophrenia patients compared to those with recurrent major depression. While the present study only explored the last month of life, some studies has suggested that women with schizophrenia may not benefit from the same prevention strategy for breast cancer screening than those without SPD^26^. This may lead to a latter care and poorer prognosis. Our database did not include cancer stage to confirm this hypothesis. Schizophrenia and bipolar disorders share common features that are not found in recurrent depression leading to less adherence to care (with impulsive behavior, treatment withdrawal, impaired cognition, addictions, psychotic and mood symptoms impairing decision-making)^27^. The increased rate of mechanical ventilation before death is probably an indicator for cardio-respiratory complications of cancer (including thromboembolic complications). The international syntheses of mortality data have concluded that the loss of life expectancy of patients with schizophrenia and bipolar disorders was due to cardiac causes in most of the cases^28,29^. This is consistent with our results and indicates the need for better cardiac prevention and care in women with schizophrenia or bipolar disorders with breast cancer. Women with recurrent major depression are older which can explain that they have the highest rate of comorbidities (> = 3 comorbidities = 26.1). However, even if patients with schizophrenia and bipolar disorders have less comorbidities (15.3 and 17.4, respectively), they have more comorbidities than controls (14.7) despite their younger age. The higher level of comorbidities does not seem to be solely related to the higher age of depressed patients and affected all psychiatric pathologies.

There are various potential explanations for our findings about palliative care and high-intensity end-of-life care in schizophrenia and bipolar disorders.

On the patient level, major depression and loneliness are frequent in women with schizophrenia and bipolar disorders and terminal cancer. In addition to the factors mentioned above, the absence of caregivers is more frequent in those women, which may also explain the higher rates of SPD women in palliative care units compared to those without SPD^30–32^.

On the care-provider level, chemotherapy may be highly toxic and require absolute observance, which is often impaired in SPD patients due to cognitive decline and opposition to care^4^. The lower imaging/endoscopy or ED/ ICU admission rate in SPD women may be explained by the diagnostic overshadowing (*i.e.,* inadequate or delayed treatment on account of the misattribution of their physical symptoms to their mental illness) that is a major concern in patients with SPD^33,34^. It should be also underlined that burnout and compassion fatigue in multidisciplinary professionals who care for those treated for cancer may impact the care, especially those delivered to women with SPD^35^.

On the health care system level, palliative care units have been designed to manage complicated situations (*e.g.*, including lack of social support/isolation, impaired autonomy that may interfere with each step of cancer care)^31,32,36^, which may explain the longer length of palliative follow-up in SPD patients. Our findings should now be completed with more qualitative approaches to better capture patient and family preferences and views.

### Limitations

Only hospitalized patients were analyzed due to data availability in the PMSI database. Yet, only 3% of patients with cancer died at home, and only 13% died in nursing homes, in France in 2013^37^. Some clinical variables were lacking in the PMSI database like psychiatric symptomatology, treatments, tobacco status and the precise cause of death.

The date of cancer diagnosis cannot be determined precisely and we used the first record of breast cancer in the hospital database as a proxy. This choice seems reasonable because these patients received palliative care for their breast cancer and it is likely that breast cancer or its complications were the cause of death. Whereas there is no reason for different PMSI coding in SPD and non-SPD women, we cannot exclude that some variable such as smoking addiction may have been more frequently coded in women with SPD than women without SPD. Women with breast cancer were included if they had at least one end-of-life inclusion criteria identified in the last three months of life, which may not be sufficient to identify the whole population. However, the medico-administrative bases are more associated with over-coding than under-coding for these codes, which are often associated with a better valuation. Another issue is that all women dying from their breast cancer should have a metastasis, which was not found in all cases. The fact that metastases were less frequently diagnosed in SPD women may indicate a shortage in health care but this hypothesis cannot be confirmed with our data. Future work should specifically explore this issue, either in terms of coding quality or as an indicator of a problem in the quality of care.

## Conclusion

Despite more palliative care and less high-intensity care in women with SPD, our findings also suggest the existence of health disparities in women with bipolar disorders and schizophrenia compared to women with recurrent major depression and without SPD. Targeted interventions may be needed for women with bipolar disorders and schizophrenia to prevent these health disparities.

## Supplementary information

Supplementary Information.

## Data Availability

Data availability is not applicable to this article due to legal restrictions imposed by the French *Agence technique de l'information sur l'hospitalisation* (*ATIH*) which restricts access to data to French hospital staff.
