# Supplementary material for: End of life breast cancer care in women with severe mental illnesses
Source: Sci Rep. 2021 May 13;11:10167. doi: 10.1038/s41598-021-89726-y (PMC8119688; doi:10.1038/s41598-021-89726-y)
Supplement: Supplementary file 1 — Supplementary Information. [file 41598_2021_89726_MOESM1_ESM.docx]

# End of life breast cancer care in women with severe mental illnesses

Running title: End-of-life and psychiatric disorders with breast cancer

FOND Guillaume (MD, PhD)^1,2,3^, PAULY Vanessa (PhD)^1,3^, DUBA Audrey, (MD)^1,2,3^, SALAS Sebastien^4^ (MD, PhD), VIPREY Marie (PharmD, PhD)^1,2^, BAUMSTARCK Karine (MD, PhD)^1^, ORLEANS Veronica (PhD)^3^, LLORCA Pierre-Michel (MD, PhD)^5^, LANCON Christophe (MD, PhD)^1,6^, AUQUIER Pascal (MD, PhD)^1,2^, BOYER Laurent (MD, PhD)^1,2,3^

1. Aix-Marseille Univ., CEReSS - Health Service Research and Quality of Life Center, Marseille, France.

2. Department of Epidemiology and Health Economics, APHM, Marseille, France.

3. Department of Medical Information, APHM, Marseille, France.

4. Department of Adult Oncology, APHM, Marseille, France.

5. CHU Clermont-Ferrand, Clermont-Ferrand, France.

6. Department of Psychiatry, APHM, Marseille, France.

*** Correspondence should be sent to: Dr Guillaume FOND**

Aix-Marseille Univ, Faculté de Médecine - Secteur Timone, EA 3279: CEReSS -Centre d'Etude et de Recherche sur les Services de Santé et la Qualité de vie, 27 Boulevard Jean Moulin, 13005 Marseille, France.

Tel: (33 6 68 10 22 58), e-mail: guillaume.fond@ap-hm.fr

| Supplementary table 1. Characteristics and between groups comparisons of the 1,742 women who died from their terminal breast cancer between 2014 and 2018 in France with diagnosis of Bipolar disorder (BD), Schizophrenia (SZ) and Recurrent major depression (RMD). | | | | | | | | | |
| --- | --- | --- | --- | --- | --- | --- | --- | --- | --- |
|  | Women with BD | | Women with SZ | | Women with RMD | | between group comparisons: p values | | |
|  | N = 287 | 0.7% | N = 380 | 1.0% | N = 1075 | 2.8% | BD vs SZ | BP vs RMD | SZ vs RMD |
|  | N | % | N | % | N | % |  |  |  |
| Age at death, years (mean [SD]) | 64.9 | [13.2] | 64.6 | [13.6] | 69.0 | [14.3] | 0.999 | < 0.001 | < 0.001 |
| Social deprivation index |  |  |  |  |  |  | 0.999 | 0.158 | 0.636 |
| More favored (Q1) | 99 | 34.5 | 121 | 31.8 | 284 | 26.4 |  |  |  |
| Favored (Q2) | 50 | 17.4 | 66 | 17.4 | 213 | 19.8 |  |  |  |
| Deprived (Q3) | 81 | 28.2 | 104 | 27.4 | 322 | 30.0 |  |  |  |
| More deprived (Q4) | 57 | 19.9 | 89 | 23.4 | 256 | 23.8 |  |  |  |
| Year of death |  |  |  |  |  |  | 0.999 | 0.999 | 0.995 |
| 2014 | 55 | 19.2 | 63 | 16.6 | 190 | 17.7 |  |  |  |
| 2015 | 59 | 20.6 | 76 | 20.0 | 247 | 23.0 |  |  |  |
| 2016 | 55 | 19.2 | 71 | 18.7 | 221 | 20.6 |  |  |  |
| 2017 | 61 | 21.3 | 85 | 22.4 | 198 | 18.4 |  |  |  |
| 2018 | 57 | 19.9 | 85 | 22.4 | 219 | 20.4 |  |  |  |
| Survival time, days (median [IQR]) | 930.0 | [343-1462] | 740.0 | [303-1330] | 962.0 | [455-1476] | 0.314 | 0.709 | 0.002 |
| Metastasis | 243 | 84.7 | 309 | 81.3 | 870 | 80.9 | 0.769 | 0.436 | 0.999 |
| Smoking addiction | 24 | 8.4 | 37 | 9.7 | 69 | 6.4 | 0.999 | 0.738 | 0.097 |
| Comorbidities |  |  |  |  |  |  |  |  |  |
| Charlson's comorbidity modified score |  |  |  |  |  |  | 0.999 | 0.001 | < 0.001 |
| 0 | 140 | 48.8 | 192 | 50.5 | 399 | 37.1 |  |  |  |
| 1-2 | 97 | 33.8 | 130 | 34.2 | 395 | 36.7 |  |  |  |
| ≥3 | 50 | 17.4 | 58 | 15.3 | 281 | 26.1 |  |  |  |
| Renal disease | 23 | 8.0 | 24 | 6.3 | 145 | 13.5 | 0.999 | 0.037 | < 0.001 |
| Rheumatologic disease | 1 | 0.4 | 4 | 1.1 | 20 | 1.9 | 0.890 | 0.194 | 0.864 |
| Peripheral Vascular disease | 7 | 2.4 | 13 | 3.4 | 67 | 6.2 | 0.999 | 0.035 | 0.116 |
| Peptic Ulcer disease | 4 | 1.4 | 1 | 0.3 | 14 | 1.3 | 0.281 | 0.999 | 0.254 |
| Hemiplegia or Paraplegia | 37 | 12.9 | 42 | 11.1 | 117 | 10.9 | 0.999 | 0.999 | 0.999 |
| Moderate or severe liver disease | 9 | 3.1 | 10 | 2.6 | 56 | 5.2 | 0.999 | 0.430 | 0.114 |
| Mild liver disease | 12 | 4.2 | 14 | 3.7 | 52 | 4.8 | 0.999 | 0.999 | 0.999 |
| AIDS/HIV | 0 | 0.0 | 4 | 1.1 | 3 | 0.3 | 0.244 | 0.999 | 0.183 |
| Diabetes with complications | 7 | 2.4 | 6 | 1.6 | 63 | 5.9 | 0.999 | 0.059 | 0.002 |
| Diabetes without complications | 43 | 15.0 | 57 | 15.0 | 209 | 19.4 | 0.999 | 0.252 | 0.162 |
| Dementia | 21 | 7.3 | 33 | 8.7 | 111 | 10.3 | 0.999 | 0.378 | 0.999 |
| Cerebrovascular disease | 22 | 7.7 | 16 | 4.2 | 93 | 8.7 | 0.170 | 0.999 | 0.014 |
| Chronic pulmonary disease | 21 | 7.3 | 35 | 9.2 | 132 | 12.3 | 0.999 | 0.054 | 0.320 |
| Congestive Heart Failure | 31 | 10.8 | 46 | 12.1 | 224 | 20.8 | 0.999 | < 0.001 | < 0.001 |
| Myocardial infarction | 6 | 2.1 | 14 | 3.7 | 54 | 5.0 | 0.696 | 0.094 | 0.863 |
| Hospital category (at last hospitalization before death) |  |  |  |  |  |  | 0.999 | 0.999 | 0.999 |
| Specialty Center | 88 | 30.7 | 111 | 29.2 | 305 | 28.4 |  |  |  |
| Nonspecialty Center | 199 | 69.3 | 269 | 70.8 | 770 | 71.6 |  |  |  |
| N: number of patients; %: percentage; IQR: interquartile range; Q: quartile (from Q1 to Q4). SD : Standard Deviation. | | | | | | | | | |

| Supplemantary table 2. Comparison of end of life palliative and high-intensity care of terminal breast cancer between women with Bipolar disorder (BD), Schizophrenia (SZ) and Recurrent major depression (RMD). | | | | | | | | | |
| --- | --- | --- | --- | --- | --- | --- | --- | --- | --- |
|  | Women with BD | | Women with SZ | | Women with RMD | | between group comparisons: p values | | |
|  | N = 287 | 0.7% | N = 380 | 1.0% | N = 1075 | 2.8% | BD vs SZ | BP vs RMD | SZ vs RMD |
|  | N or Median | % or [IQR 95%] | N or Median | % or [IQR 95%] | N or Median | % or [IQR 95%] | |  |  |
| Palliative care |  |  |  |  |  |  |  |  |  |
| Palliative care in the last 31 days of life | 225 | 78.4 | 301 | 79.2 | 891 | 82.9 | 0.999 | 0.153 | 0.221 |
| Duration (days) between the first palliative care and death (for patients with palliative care) | 24.5 | [9-56] | 26.0 | [13-66] | 29.0 | [11-93] | 0.765 | 0.059 | 0.817 |
| High-intensity end-of-life care |  |  |  |  |  |  |  |  |  |
| Intrahospital chemotherapy in the last 14 days of life | 42 | 14.6 | 36 | 9.5 | 116 | 10.8 | 0.210 | 0.286 | 0.999 |
| Mechanical ventilation in the last 31 days of life | 40 | 13.9 | 55 | 14.5 | 101 | 9.4 | 0.999 | 0.046 | 0.045 |
| Blood transfusion in the last 31 days of life | 43 | 15.0 | 37 | 9.7 | 124 | 11.5 | 0.327 | 0.443 | 0.999 |
| Surgery in the last 31 days of life | 44 | 15.3 | 38 | 10.0 | 97 | 9.0 | 0.126 | 0.011 | 0.999 |
| Imaging/ Endoscopy in the last 31 days of life | 195 | 67.9 | 251 | 66.1 | 705 | 65.6 | 0.999 | 0.999 | 0.999 |
| At least one ED admission in the last 31 days of life | 102 | 35.5 | 176 | 46.3 | 432 | 40.2 | 0.207 | 0.999 | 0.338 |
| At least one ICU admission in the last 31 days of life | 24 | 8.4 | 31 | 8.2 | 62 | 5.8 | 0.999 | 0.352 | 0.999 |
| More than one admission in complete acute care unit in the last 31 days of life | 135 | 47.0 | 171 | 45.0 | 486 | 45.2 | 0.999 | 0.999 | 0.999 |
| N: number of patients; %: percentage; IQR: interquartile range.  SD : Standard Deviation. | | | | | | | | | |

Appendix A1. Definitions used to derive each outcome.

| Outcomes | Definitions |
| --- | --- |
| Palliative-care unit admission in the last 31 days of life | At least one hospitalization with authorization of palliative unit or bed care or ICD-10 code of palliative care: Z515 for PMSI-MCO in the last 31 days of life |
| Last intra-hospital chemotherapy < 14 days from death | At least one hospitalization ending within 14 days preceding death, with diagnostic code Z511, Z512, Z082 (ICD 10th version code) |
| Mechanical ventilation in the last 31 days of life | Presence of at least one of the following codes of medical procedure* in the last 31 days of life: GLLD001 - GLLD002 -GLLD003 - GLLD004 - GLLD006 - GLLD007 - GLLD008 - GLLD009 - GLLD010 - GLLD011 - GLLD012 - GLLD013 - GLLD015 - GLLD019 - GDLD001 - GELD002 - GELD003 - GELD004 - GELE003 - GELE004 |
| Surgery in the last 31 days of life | Presence of at least one of codes of surgery procedure within 31 days preceding death as defined by the CCAM classification * |
| Endoscopy in the last 31 days of life | Presence of at least one of codes of endoscopy procedure within 31 days preceding death as defined by the CCAM classification * |
| Imaging in the last 31 days of life | Presence of at least one of codes of imaging procedure within 31 days preceding death as defined by the CCAM classification * |
| At least one emergency room admission in the last 31 days of life | One or more than one admission in an emergency room admission (admission codification =85) within 31 days preceding death |
| At least one reanimation/ICU admission in the last 31 days of life | One or more than one admission in an acute care hospital (MCO) within 31 days preceding death |
| More than one admission in acute care unit in the last 31 days of life | More than one admission in an acute care hospital (MCO) within 31 days preceding death |

*codes of medical procedure based on the French Common Classification of Medical Procedures (Classification Commune des Actes Médicaux or CCAM): <https://www.ameli.fr/accueil-de-la-ccam/index.php>

** <https://www.atih.sante.fr/nomenclatures-de-recueil-de-l-information/autorisations-des-unites-medicales>
